# Effects of Finger Tapping Frequency on Regional Homogeneity of Sensorimotor Cortex

**DOI:** 10.1371/journal.pone.0064115

**Published:** 2013-05-16

**Authors:** Yating Lv, Daniel S. Margulies, Arno Villringer, Yu-Feng Zang

**Affiliations:** 1 Max Planck Institute for Human Cognitive and Brain Sciences, Leipzig, Germany; 2 Center for Cognition and Brain Disorders, Affiliated Hospital, Hangzhou Normal University, Hangzhou, China; 3 Berlin School of Mind and Brain, Humboldt Universität, Berlin, Germany; 4 State Key Laboratory of Cognitive Neuroscience and Learning, Beijing Normal University, Beijing, China; 5 Zhejiang Key Laboratory for Research in Assessment of Cognitive Impairments, Hangzhou, China; University of Massachusetts Medical School, United States of America

## Abstract

Resting-state functional magnetic resonance imaging (RS-fMRI) has been widely used to investigate temporally correlated fluctuations between distributed brain areas, as well as to characterize local synchronization of low frequency (<0.1 Hz) spontaneous fMRI signal. Regional homogeneity (ReHo) was proposed as a voxel-wise measure of the synchronization of the timecourses of neighboring voxels and has been used in many studies of brain disorders. However, the interpretation of ReHo remains challenging because the effect of high frequency task on ReHo is still not clear. In order to investigate the effect of a high-frequency task on the modulation of local synchronization of resting-state activity, we employed three right-finger movement scanning sessions: slow-event related (‘Slow’), fast-event related (‘Fast’), and continuous finger pressure (‘Tonic’), from 21 healthy participants and compared the ReHo of the three task states with that of resting-state (‘Rest’). In the contralateral sensorimotor cortex, ‘Slow’ task state showed greater ReHo than ‘Rest’ in low frequency band (0–0.08Hz) fMRI signal, but lower ReHo in high frequency band (0.08–1.67 Hz); ‘Fast’ task state showed lower ReHo than ‘Rest’ in both the low and high frequency band; ‘Tonic’ state did not show any significant difference compared to ‘Rest’. The results in the contralateral sensorimotor cortex suggest that local synchronization of BOLD signal varies with different finger tapping speed. In the ipsilateral sensorimotor cortex, all the three task states had lower ReHo than the ‘Rest’ state both in the low and high frequency, suggesting a similar effect of fast and slow finger tapping frequencies on local synchronization of BOLD signal in the ipsilateral motor cortex.

## Introduction

Resting-state functional magnetic resonance imaging (RS-fMRI) is widely used to investigate temporally correlated fluctuations (otherwise known as “functional connectivity”) between distributed brain areas [1]. For example, functional connectivity exists between the left and right motor cortices in healthy normal individuals [1], [2], [3]. While functional connectivity analysis measures the synchronization of the timecourses between distinct brain areas, a few different methods have been proposed to characterize the local synchronization of the RS-fMRI signal [4], [5], [6]. For example, Li and colleagues proposed the cross-correlation coefficients of spontaneous low frequency (COSLOF) approach to measure the synchronization of the timecourses of voxels within the hippocampus and found that COSLOF was a sensitive index for Alzheimer disease (AD) [5]. Another method, regional homogeneity (ReHo), was proposed as a voxel-wise measure of the synchronization of the timecourses of neighboring voxels [6]. Several RS-fMRI studies have demonstrated brain regions that consistently show higher ReHo than others [7], [8], [9], the pattern of which is consistent with the default mode network during resting-state revealed by positron emission tomography (PET) study [10]. With the advantage of having easy implementation, ReHo analysis has been used to investigate functional modulations in the resting-state in many brain disorders including schizophrenia [11], attention deficit hyperactivity disorder (ADHD) [12], AD [13], Parkinson's disease [14], epilepsy [15], and autism [16]. For example, decreased ReHo of the RS-fMRI BOLD in the posterior cingulate cortex (PCC) in AD patients has been reported to be highly consistent with the decreased glucose metabolism found in AD patients by PET [13]. From these results, higher ReHo of BOLD signal may indicate higher synchronization of local field potential of neuronal activity in the human brain.

While using ReHo to measure the local synchronization shows promising clinical usefulness, the interpretation of altered local synchronization remains unclear. In a ReHo study of the primary sensorimotor cortex (PSMC), Zang and colleagues found higher ReHo during a motor task state than resting-state, and vice versa, higher ReHo in the default mode network during resting-state than the motor task state [6]. These patterns are very consistent with the pattern of activation and deactivation in traditional task fMRI studies. A reasonable interpretation is that an evoked activity is associated with increased ReHo. However, two important issues were not addressed in Zang and colleagues' study [6]. Firstly, the task state was part of a slow event-related design with an averaged inter-stimulus interval (ISI) of 15 s. Therefore, the interpretation of the ReHo results could not be generalized to a fast event-related design or to some clinical conditions which have high frequency neuronal activity, e.g., some seizures in epilepsy or tremor in Parkinson's disease. Secondly, the left and right finger tapping tasks were incorporated into one session in a pseudorandom order. Therefore, to calculate the ReHo value for left and right finger tapping tasks separately, the timecourses had to be truncated and then re-connected to acquire two time serials for each task [6]. This concatenation procedure could be problematic because the hemodynamic response may have not returned to the baseline after about 15 s. Consequently, there are at least two questions remain unresolved: 1) Can the results from Zang and colleagues' ReHo study [6] be generalized to a fast event-related design? 2) What will happen to the ReHo in the ipsilateral PSMC in a fast motor task state?

To address these questions, in the current study, we used four scanning sessions: one resting session and three finger movement sessions with different rates of movement to investigate ReHo changes in the sensorimotor cortex in a group of healthy participants. We used fast acquisition in order to adequately address the questions of the current study. For the low rate finger movement condition, we predict that the low frequency synchronization will increase as compared to resting-state in the primary motor cortex. Inversely, for the fast finger tapping condition, the ReHo change is not clear and therefore is a more explorative question.

## Materials and Methods

### Participants

Twenty-one graduate and postgraduate students (age = 19–26 years; mean = 22.5 years; 7 males and 14 females) participated in the current study. All participants were right-handed and had no history of neurological, psychiatric, or movement disorders. The Institutional Review Board of State Key Lab for Cognitive Neuroscience and Learning, Beijing Normal University (BNU) approved this study, and all participants gave informed written consent.

### Data acquisition

Functional imaging data were acquired using a SIEMENS 3T TRIO scanner, with a standard head coil, located in the BNU Brain Imaging Center. Four scanning sessions (5 axial slices covering the sensorimotor cortex, TR = 300 ms, thickness/gap = 5/1 mm, total volumes = 1010) were obtained: 1) Rest (RS-fMRI) scanning: The participants were instructed to keep as still as possible and not to think about anything in particular; 2) Tonic pressing task-state fMRI scanning: The participants were instructed to hold a clamp with the right thumb and index fingers without loosing it during this session; 3) Fast event-related task-state fMRI scanning: The participants completed a fast event-related task. Inter-trial intervals (ITI) varied between 3.6 and 6.6 s (mean ITI = 5.1 s) in a pseudorandom order. On each trial in this task, a red star flashed at a frequency of 2 Hz, four times, and participants were instructed to press a key with their right index finger following the red star flashing frequency; 4) Slow event-related task-state fMRI scanning: The task was the same as the task of session 3, but with ITI between 13.5 and 16.5 s (mean ITI = 15 s). The four states were labeled ‘Rest’, ‘Tonic’, ‘Fast’ and ‘Slow’ respectively. The order of 1) and 2), the order of 3) and 4) were counterbalanced across participants. We used fast scanning (TR = 300 ms) because it allowed for better investigation of the frequency effect (below 0.1 Hz vs. full frequency band) of the RS-fMRI signal. In addition, it has been indicated that the cardiac pulse (usually 1.2 Hz) affects the BOLD signal (here, about 3.3 Hz with TR = 300 ms), and thus could be largely reduced by band pass (0.01–0.08 Hz) filtering [17], [18].

Two anatomical sessions were obtained: 1) Five axial T1 slices that covered the sensorimotor cortex: The slice position was the same as for the functional images. The other parameters were slice thickness/gap = 5/1 mm, TR = 250 ms, TE = 2.46 ms, matrix = 448×512, and FOV = 17.5×20 cm; 2) 3D high-resolution anatomical images of the entire brain were obtained with the following parameters: TR = 2530 ms, TE = 3.39 ms, matrix size = 256×256, 128 sagittal slices.

### Data Analysis

#### Data preprocessing

Preprocessing of fMRI data was performed using statistical parametric mapping (SPM5, http://www.fil.ion.ucl.ac.uk/spm). Slice timing and head motion correction were performed. No participant's head motion exceeded 1 mm of displacement or 1 degree of rotation. The fMRI data was then re-sampled (3×3×3 mm^3^). Head motion effect (using six motion parameters: three rigid body translations and three rotations) was also regressed out from the fMRI data. The Resting-State fMRI Data Analysis Toolkit (REST, http://www.restfmri.net) [19] was then used to remove the linear trend of time courses and for band pass filtering, for which we used four different frequency bands: 0.01–0.08 Hz which has been commonly used in resting-state data processing [1], [17], 0–0.08 Hz, which we used to study the very low frequency range's effect, 0.08–1.67 Hz, which we used to study the high frequency effect, and whole frequency band (without filtering). We then calculated ReHo after bandpass filtering using REST.

#### Regional homogeneity (ReHo)

We used regional homogeneity (ReHo) method [6], in which Kendall's coefficient of concordance (KCC) was used to measure the functional synchronization of the timecourses of neighboring voxels as follows:
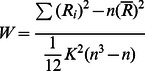
(1) where *W* is the KCC among given voxels, ranging from 0 to 1; *R_i_* is the sum rank of the *i*th time point; 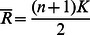 is the mean of the *R_i_*'s; *K* is the number of time series within a measured cluster (K = 7, 19, and 27, respectively. 27 in the current study); and *n* is the number of ranks.

We calculated four ReHo values (band pass range: 0.01–0.08 Hz, 0–0.08 Hz, 0.08–1.67 Hz and without filtering) for each of the four conditions. All the resultant ReHo maps were aligned to individual 3D images via T1 images and subsequently spatially normalized to the Montreal Neurology Institute (MNI) template before being smoothed with a 6-mm FWHM isotropic Gaussian Kernel using SPM5 (http://www.fil.ion.ucl.ac.uk/spm).

For each participant, we acquired one resting-state session and three task sessions with TR = 300 ms and 5 axial slices covering the sensorimotor cortex. As the location of the five slices did not completely overlap across participants, we made an intersection mask which included common brain areas scanned for all participants. Paired *t*-tests were then performed to compare the ReHo of the three task states with that of ‘Rest’ state within the intersection mask. The resultant T-map was threshold with *p*<0.05 (uncorrected for multiple comparisons) and also *p*<0.05 (Alphasim correction for multiple comparisons (http://afni.nimh.nih.gov/afni/doc/manual/AlphaSim [19], [20])).

#### Task-evoked activation analysis

After the head motion correction, the functional images of ‘Fast’ and ‘Slow’ tasks were aligned to individual 3D images via T1 images and subsequently spatially normalized to the MNI template and then smoothed with a 6-mm FWHM isotropic Gaussian Kernel using SPM5 (http://www.fil.ion.ucl.ac.uk/spm). The resultant data was further processed to generate the individual- and group-level activation maps by using a general linear model: for each task the sequence of temporal events was modeled with an SPM5 design matrix and convolved with the canonical hemodynamic response function (HRF). The six individual movement parameters were included as confounding variables. The individual contrast was calculated for each participant during ‘Fast’ and ‘Slow’ finger tapping conditions. Voxel-wise one-sample *t*-tests against the null hypothesis of zero magnitude were used to calculate group level map for both the ‘Fast’ and ‘Slow’ finger tapping tasks.

## Results

### ‘Fast’ and ‘Slow’ task activations

**Figure 1:**
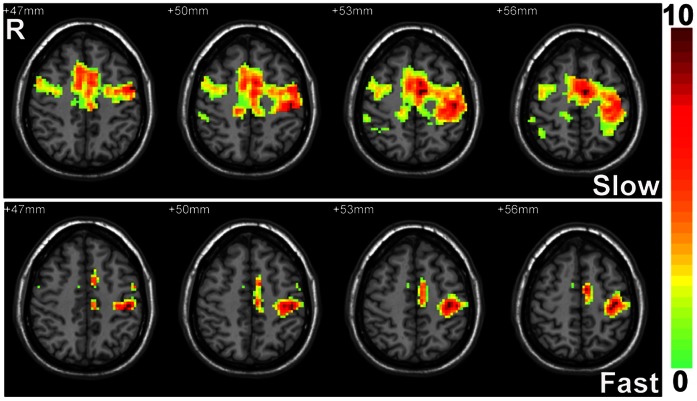
The group activation map of ‘Slow’ and ‘Fast’ task state. (*p*<0.05, FDR correction, *k* = 10 voxels).

As expected, one-sample *t*-tests (*p*<0.05, FDR correction, *k* = 10 voxels) showed that both ‘Fast’ and ‘Slow’ right index finger tapping tasks activated the left primary sensorimotor cortex (PSMC). In addition, the ‘Fast’ task activated the left supplementary motor area (SMA). The ‘Slow’ task activated bilateral SMA, right premotor area, and bilateral parietal area (Fig. 1).

### Comparison of ReHo between ‘Rest’ state and three task states

**Figure 2:**
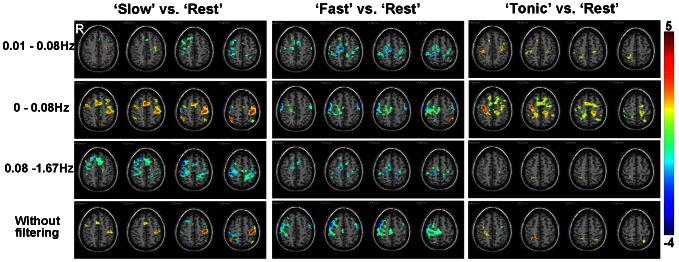
ReHo comparison results between different states. The results comparing the ReHo of the three task states with that of ‘Rest’ state with different band pass filtering (0.01–0.08 Hz, 0–0.08 Hz, 0.08–1.67 Hz) and without filtering. Warm color represents higher ReHo than ‘Rest’ state, cold color represents lower ReHo than ‘Rest’ state (*p*<0.05, uncorrected).

The comparison results among the different conditions were shown in Table 1, Table 2, Table 3 and Figure 2. Table 1 was just the rough summary of ReHo comparison results among different states, which were more complicated, as shown in Figure 2.

**Table 1:** A rough summary of ReHo comparison results between three task states and resting-state in bilateral sensorimotor cortex.

Filtering band range (Hz)	Slow vs. Rest		Fast vs. Rest		Tonic vs. Rest	
	Cont^a^	Ipsi^b^	Cont	Ipsi	Cont	Ipsi
0.01–0.08	**+**	**--**	**--**	**--**		
0–0.08	**++**	**--**	**--**	**--**		**-**
0.08–1.67	**--**	**--**	**-**	**-**		
no filter	**++**	**--**	**-**	**--**		**-**

a Cont: Contralateral hemisphere to the tapping finger (Left hemisphere);

b Ipsi: Ipsilateral hemisphere to the tapping finger (Right hemisphere);

++/--: Significant increase or decrease (*p*<0.05, Alphasim correction);

+/-: Significant increase or decrease (*p*<0.05, uncorrected).

**Table 2:** ReHo values in bilateral sensorimotor cortex across participants: mean value (standard deviation) in different conditions of ‘Slow vs. Rest’.

Filtering band range(Hz)	Slow vs. Rest mean (SD^c^)							
	Cont^a^				Ipsi^b^			
	Slow	Rest	Diff^d^	Tonic	Slow	Rest	Diff^d^	Tonic
0.01–0.08	0.31(0.11)	0.26(0.09)	0.05(0.07)	0.32(0.13)	0.38(0.08)	0.49(0.12)	−0.11(0.09)	0.47(0.09)
0–0.08	0.40(0.10)	0.32(0.08)	0.08(0.07)	0.34(0.08)	0.32(0.11)	0.35(0.12)	−0.03(0.05)	0.33(0.13)
0.08–1.67	0.23(0.05)	0.25(0.05)	−0.02(0.05)	0.24(0.05)	0.26(0.06)	0.34(0.09)	−0.07(0.07)	0.32(0.07)
no filter	0.20(0.04)	0.18(0.04)	0.02(0.02)	0.18(0.03)	0.18(0.05)	0.20(0.05)	−0.02(0.03)	0.19(0.05)

a Cont: Contralateral hemisphere to the tapping finger (Left hemisphere);

b Ipsi: Ipsilateral hemisphere to the tapping finger (Right hemisphere);

c SD: Standard deviation;

d Diff: ReHo Difference in each ROI between ‘Slow’ and ‘Rest’ states. The difference value was first calculated within subject and then averaged across subjects.

We made cubic region of interests (ROIs) (27 voxels) in bilateral primary sensorimotor cortex (PSMC). The bilateral peak voxels in different conditions, which showed significant difference in ‘Slow vs. Rest’ (Figure 2), were picked as center for cubic ROIs. For each participant, the mean ReHo value of each mask for ‘Slow’, ‘Rest’ and ‘Tonic’ states, as well as difference ReHo value between ‘Slow’ and ‘Rest’ states (Diff) were calculated. The group mean values and standard deviations were then calculated across subjects as showed in the table.

**Table 3:** ReHo values in bilateral sensorimotor cortex across participants: mean value (standard deviation) in different conditions of ‘Fast vs. Rest’.

Filtering band range(Hz)	Fast vs. Rest mean (SD^c^)							
	Cont^a^				Ipsi^b^			
	Fast	Rest	Diff^d^	Tonic	Fast	Rest	Diff^d^	Tonic
0.01–0.08	0.29(0.07)	0.36(0.10)	−0.07(0.09)	0.37(0.09)	0.21(0.08)	0.27(0.09)	−0.06(0.05)	0.28(0.11)
0–0.08	0.31(0.06)	0.34(0.06)	−0.03(0.03)	0.33(0.07)	0.29(0.09)	0.35(0.12)	−0.06(0.06)	0.32(0.12)
0.08–1.67	0.20(0.04)	0.22(0.05)	−0.03(0.04)	0.23(0.05)	0.16(0.05)	0.18(0.05)	−0.03(0.03)	0.18(0.05)
no filter	0.19(0.02)	0.20(0.02)	−0.01(0.01)	0.19(0.02)	0.26(0.03)	0.28(0.03)	−0.02(0.02)	0.28(0.04)

a Cont: Contralateral hemisphere to the tapping finger (Left hemisphere);

b Ipsi: Ipsilateral hemisphere to the tapping finger (Right hemisphere);

c SD: Standard deviation.

d Diff: ReHo Difference in each ROI between ‘Fast’ and ‘Rest’ states. The difference value was first calculated within subject and then averaged across subjects.

We made cubic region of interests (ROIs) (27 voxels) in bilateral primary sensorimotor cortex (PSMC). The bilateral peak voxels in different conditions, which showed significant difference in ‘Fast vs. Rest’ (Figure 2), were picked as center for cubic ROIs. For each participant, the mean ReHo value of each mask for ‘Fast’, ‘Rest’ and ‘Tonic’ states, as well as difference ReHo value between ‘Fast’ and ‘Rest’ states (Diff) were calculated. The group mean values and standard deviations were then calculated across subjects as showed in the table.

1. **‘Slow’ vs. ‘Rest’.** In the contralateral PSMC, ‘Slow’ had higher ReHo than ‘Rest’ with 0.01–0.08 Hz filtering (*p*<0.05), 0–0.08 Hz filtering and without filtering (*p*<0.05, corrected), while ‘Slow’ had lower ReHo with 0.08–1.67 Hz band pass filtering (*p*<0.05, corrected). It should be noticed here that the very low frequency (<0.01 Hz) contributed substantially to the whole frequency band results (without filtering). In the ipsilateral PSMC, ‘Slow’ had lower ReHo than the ‘Rest’ state (*p*<0.05, corrected) with different band pass filtering (Fig. 2).
2. **‘Fast’ vs. ‘Rest’.** In the contralateral PSMC, ‘Fast’ task state had lower ReHo than ‘Rest’ state with a band pass filter of 0.01–0.08 Hz and 0–0.08 Hz (*p*<0.05, corrected), as well as with 0.08–1.67 Hz band pass filtering and without filtering (*p*<0.05). In the ipsilateral PSMC, ‘Fast’ also had lower ReHo than ‘Rest’ with a band pass filter of 0.01–0.08 Hz, 0–0.08 Hz and without filtering (*p*<0.05, corrected), as well as with 0.08–1.67 Hz band pass filtering (*p*<0.05). (Fig. 2)
3. **‘Tonic’ vs. ‘Rest’.** ‘Tonic’ task state had lower ReHo than ‘Rest’ state (*p*<0.05) with a band pass filter of 0–0.08 Hz, and without filtering in the ipsilateral PSMC. There was no significant ReHo difference in the contralateral PSMC (Fig. 2).

## Discussion

### Contralateral PSMC

**Figure 3:**
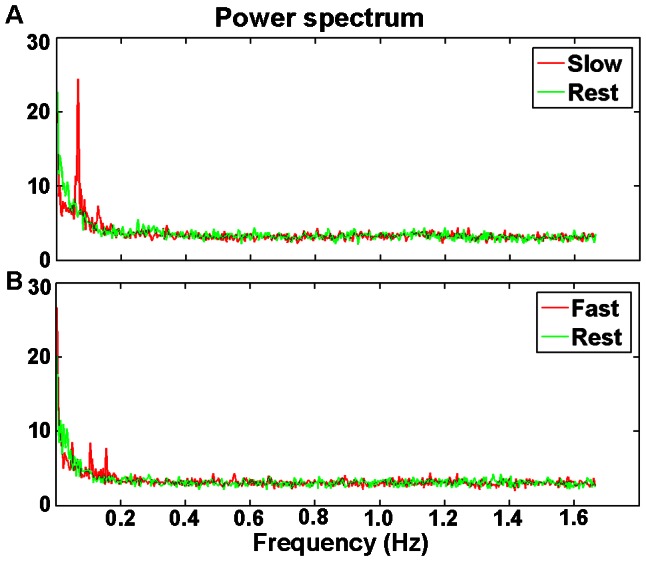
Power spectrum. Power spectrum of peak voxel which showed higher ReHo in the ‘Slow’ compared with ‘Rest’ state (with 0–0.08 Hz bandpass filtering). A: ‘Slow’ vs. ‘Rest’ power spectrum; B: ‘Fast’ vs. ‘Rest’ power spectrum.

1. **‘Slow’ vs. ‘Rest’.** ‘Slow’ had higher ReHo than the ‘Rest’ with 0.01–0.08 Hz, 0–0.08 Hz, and without filtering. The frequency of the ‘Slow’ task in the current study was approximately 0.067 Hz (ITI varied from 13.5 s to 16.5 s, mean ITI = 15 s). Therefore, the increased ReHo, or local synchronization, may imply that low frequency activity was increased, as was evident from the power spectrum of ‘Slow’ task state of a representative voxel (Fig. 3A), while no increased low frequency activity in power spectrum of ‘Fast’ state (Fig. 3B). The increased ReHo in the contralateral PSMC in ‘Slow’ compared to ‘Rest’ state was consistent with the previous ReHo study [6], indicating an increased local synchronization of low frequency fluctuations. It should be noted that no band pass filtering was used in that study. These results indicate that ‘Slow’ finger movement may affect the synchronization of BOLD signal in a wide range of frequency bands, the very low frequency (<0.01 Hz) contributed substantially to the whole frequency band results (without filtering). In RS-fMRI studies, 0.01–0.08 Hz is widely used as the window for band pass filtering. The current results suggest that frequencies below and above this conventional frequency band might also be useful for revealing the spontaneous neural activity. In the future, we hope these results could partly help understand the ReHo changes in both physiology and pathophysiology disorders with complicated abnormal spontaneous brain activity, e.g., epilepsy and movement disorders. However, the underlying mechanisms need to be investigated in future studies.
2. **‘Fast’ vs. ‘Rest’.** ‘Fast’ task state had lower ReHo than ‘Rest’ state with all 4 types of band pass filtering. Brain activation elicited by such ‘Fast’ task have been reported by many task-state fMRI studies [21], [22]. For example, Rao et al. used a block design with finger-tapping task to study the “rate effect” on signal change in the primary motor cortex. The probability of “activation” in a block design is the ratio of mean signal difference between the task block and the rest block and the total variance within blocks (see the formula of *t*-test). It means that the variance with block is taken as “noise”. The decreased ReHo during ‘Fast’ finger tapping task in current study may due to increased high frequency “noise”. These results could help interpret resting-state fMRI findings by ReHo analysis. For example, tremor may be considered to be a fast event-related task and might induce decreased ReHo in a specific brain area where the tremor is generated.
3. **‘Tonic’ vs. ‘Rest’.** There was no significant difference between ‘Tonic’ task state and ‘Rest’ state in the contralateral PSMC. The data acquisition order of ‘Rest’ and ‘Tonic’ state were counterbalanced across participants in our study, and little strength was needed to hold the clamp with the right thumb and index fingers without loosing it during ‘Tonic’ session. These may account for the lack of a significant difference between ‘Tonic’ and ‘Rest’ states. Other paradigms using different tonic tasks (e.g., bigger finger force) would be helpful to resolve this potential issue.

### Ipsilateral PSMC

All the three task states had lower ReHo than the ‘Rest’ state in ipsilateral PSMC. In previous study it was reported that the motor cortex ipsilateral to the moving hand revealed reduced hemodynamic responses during finger tapping, which might indicate that the ipsilateral PSMC is decoupled during movement of the ipsilateral hand [23]. It has also reported that for the right-handed person, the dominant contralateral PSMC (left) inhibits the ipsilateral PSMC more effectively than vice versa [24], [25]. The lower ReHo or local synchronization in task state than the ‘Rest’ state in the current study also supports this inhibition hypothesis in previous studies [23]. ReHo could be an effective tool to detect this inhibition in future studies.

Our current results in the ipsilateral PSMC seem to contradict those of Zang and colleagues’ [6], in which increased ReHo was found in the ipsilateral PSMC. One possible reason might be the difference in experimental design: the current study used only unilateral finger movements, whereas the Zang et al. used a slow event-related design with alternate left and right finger movements. The hemodynamic BOLD signal response elicited by the alternating left and right finger movements may have a temporal overlap on each other because the mean ISI in that study was approximately 15 s: that may not be long enough to allow the hemodynamic response to go back to baseline. Zang et al. also divided the timecourse into 12 s segments, each representing an event, and then concatenated 10 segments to form the timecourses of left finger movement and right finger movement, respectively. To investigate the potential confounding effect of such concatenated segments, we randomly picked 13 trials (12 s segment for each trial) in ‘Slow’ session, and then truncated and concatenated the 13 segments to generate a new timecourse as did in Zang et al. [6]. We also acquired the same length from the ‘Rest’ session as did in Zang et al. [6]. Then we compared the ReHo between the concatenated ‘Slow’ and ‘Rest’ sessions. The results were very similar to those without truncation: ‘Slow’ had lower ReHo in ipsilateral PSMC than ‘Rest’ state. In other words, the increased ReHo in the ipsilateral PSMC during task in Zang and colleagues’ study is unlikely to be due to the truncation effect, rather, a possible explanation would be the left finger tapping and right finger tapping tasks in a single session may have significant temporal interaction in the ipsilateral primary sensorimotor cortex, even in a slow event-related design. Such temporal interaction effect may not be that big in the contralateral primary sensorimotor cortex.

## Conclusions

In this study, we demonstrated that finger tapping frequency has different effects on ReHo, or local synchronization, of the sensorimotor cortex: in the contralateral PSMC, the BOLD signal local synchronization went up in low frequency band but went down in high frequency band during the ‘Slow’ task state compared to ‘Rest’ state, while the local synchronization went down in both the high and low frequency band during the ‘Fast’ task state; in the ipsilateral PSMC, the BOLD signal local synchronization went down in both the high and low frequency band during all three task states (‘Slow’, ‘Fast’, and ‘Tonic’) compared to ‘Rest’ state. These results could help understand the complexity of BOLD signal.
